# Anti-atrial Fibrillation Effects of Pulmonary Vein Isolation With or Without Ablation Gaps: A Computational Modeling Study

**DOI:** 10.3389/fphys.2022.846620

**Published:** 2022-03-17

**Authors:** Ze Jin, Inseok Hwang, Byounghyun Lim, Oh-Seok Kwon, Je-Wook Park, Hee-Tae Yu, Tae-Hoon Kim, Boyoung Joung, Moon-Hyoung Lee, Hui-Nam Pak

**Affiliations:** Yonsei University College of Medicine, Yonsei University Health System, Seoul, South Korea

**Keywords:** atrial fibrillation, computational modeling, pulmonary vein, gap, dominant frequency

## Abstract

**Background:**

Although pulmonary vein isolation (PVI) gaps contribute to recurrence after atrial fibrillation (AF) catheter ablation, the mechanism is unclear. We used realistic computational human AF modeling to explore the AF wave-dynamic changes of PVI with gaps (PVI-gaps).

**Methods:**

We included 40 patients (80% male, 61.0 ± 9.8 years old, 92.5% persistent AF) who underwent AF catheter ablation to develop our realistic computational AF model. We compared the effects of a complete PVI (CPVI) and PVI-gap (2-mm × 4) on the AF wave-dynamics by evaluating the dominant frequency (DF), spatial change of DF, maximal slope of the action potential duration restitution curve (Smax), and AF defragmentation rate (termination or change to atrial tachycardia), and tested the effects of additional virtual interventions and flecainide on ongoing AF with PVI-gaps.

**Results:**

Compared with the baseline AF, CPVIs significantly reduced extra-PV DFs (*p* < 0.001), but PVI-gaps did not. COV-DFs were greater after CPVIs than PVI-gaps (*p* < 0.001). Neither CPVIs nor PVI-gaps changed the mean Smax. CPVIs resulted in higher AF defragmentation rates (80%) than PVI-gaps (12.5%, *p* < 0.001). In ongoing AF after PVI-gaps, the AF defragmentation rates after a wave-breaking gap ablation, extra-PV DF ablation, or flecainide were 60.0, 34.3, and 25.7%, respectively (*p* = 0.010).

**Conclusion:**

CPVIs effectively reduced the DF, increased its spatial heterogeneity in extra-PV areas, and offered better anti-AF effects than extra-PV DF ablation or additional flecainide in PVI-gap conditions.

## Introduction

Catheter ablation is the most effective rhythm control method for atrial fibrillation (AF; Turagam et al., 2019). The cornerstone of AF catheter ablation (AFCA) is pulmonary vein isolation (PVI; Oral et al., 2006). However, even after AFCA with an adequate PVI, the AF recurrence rate is 40–50% within 5 years (Saguner et al., 2018). In patients with a post-AFCA recurrence during a repeat procedure, the pulmonary vein (PV) reconnection rate, which is the leading cause of recurrence, reaches 37–95% (Lin et al., 2015). PV reconnections are due to the technical limitations of the PVI durability (Arujuna et al., 2012). On the other hand, extra-PV triggers are also an important AF mechanism in AF patients with significant atrial remodeling (Velagapudi et al., 2013). Because AF is a progressive, degenerative disease, an empirical extra-PV ablation has been attempted, especially in patients with persistent or long-standing AF (Haissaguerre et al., 2005). Nevertheless, there is no evidence that an empirical extra-PV linear, electrogram-guided, or rotor ablation has a rhythm outcome superior to a complete PVI (CPVI) alone for ablating persistent AF (Verma et al., 2015). Indeed, the mechanism by which a CPVI and additional extra-PV ablation provide equivalent rhythm control is unknown. Little is known about the optimal procedure for AF patients with recurrence and an incomplete PVI (Park et al., 2019).

Therefore, this study used realistic human AF computational modeling to explore how a CPVI affects the extra-PV AF wave-dynamics and how a PVI-gap affects the recurrence mechanism. Current AF computational modeling takes advantage of recent improvements in the computational power to precisely simulate human AF by applying a personalized anatomy, histology, and electrophysiology (Lim et al., 2020a). Various virtual interventions and virtual anti-arrhythmic drug tests have become possible using controlled *in silico* conditions (Shim et al., 2017; Kim et al., 2019). In this study, we hypothesized that a CPVI would affect the extra-PV AF wave-dynamics. In addition, we evaluated the effects of a PVI with-gaps on the AF maintenance and compared the interventions and drug challenges to find the optimal anti-AF outcomes.

## Materials and Methods

### Ethical Approval

The study protocol followed the Declaration of Helsinki and was approved by the Institutional Review Board of Severance Cardiovascular Hospital, Yonsei University Health System. All participants were included in the Yonsei AF Ablation Cohort Database (ClinicalTrials.gov Identifier: NCT02138695) and provided written informed consent for us to use their cardiac CT images and clinical electrophysiological mapping data for computational modeling studies.

### 3D Computational Model of the Left Atrium

**Figure 1:**
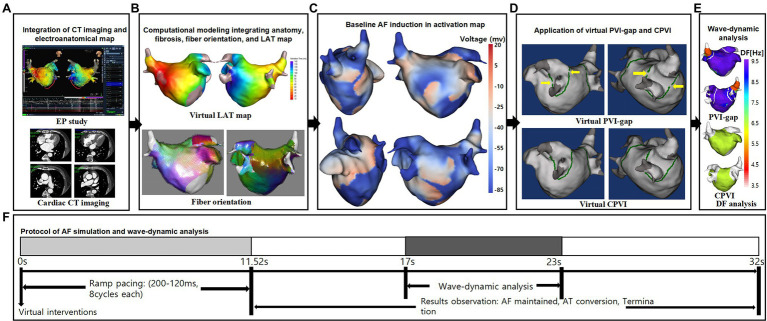
Study protocol of the computational atrial modeling and AF simulation. **(A)** CT merged 3D-clinical electroanatomical map. **(B)** Clinical map integrated computational modeling. **(C)** Baseline AF induction in activation map. **(D)** Application of CPVI and PVI-gap. The yellow arrows indicate gaps in the PVI. **(E)** DF-based wave-dynamics analyses. **(F)** Study protocol. CT indicates computed tomography; EP, electrophysiology; LAT, local activation time; PVI-gap, pulmonary vein isolation with gap; CPVI, complete pulmonary vein isolation; DF, dominant frequency; AF, atrial fibrillation; AT, atrial tachycardia.

Figure 1 illustrates the protocol for the computational atrial modeling and AF simulations used in this study. We developed an ionic currents model according to the human atrial action potential model proposed by Courtemanche et al. (1998). For AF atrial ionic remodeling, the sodium current (I_Na_), transient outward potassium current (I_to_), L-type calcium current (I_CaL_), and ultrarapid outward current (I_Kur_) decreased by 10, 70, 50, and 50%, respectively, and the inwardly rectifying potassium current (I_K1_) and Na^+^/Ca^2+^ exchanger (I_NCX_) increased by 100 and 40%, respectively (Lee et al., 2016). The surface of the left atrial (LA) 3D model was composed of triangular meshes containing 400,000–500,000 geometric elements, and the mean distance between adjacent elements was 235.1 ± 32.1 μm. Interpolated voltage data were generated from bipolar electrograms recorded from >500 points on the atrial surface using a circular mapping catheter and CT images (Figure 1A). Artifact caused by the patient’s breathing was removed by trimming the ostial positions on the PVs and mitral valve. The coordinates of the electroanatomical map (NavX, Abbott, Inc., Chicago, IL, United States; CARTO System, Biosense Webster, Diamond Bar, CA, United States) were precisely aligned with the patients’ clinical heart CT images, and then the registration between the electroanatomical maps and clinical CT data was completed. We used the inverse distance weighting method (Ugarte et al., 2015) to represent the interpolation of the electroanatomical map values during the simulation procedures. Our graphical user interface software (Model: SH01, CUVIA ver. 2.5; Laonmed Inc., Seoul, Korea) integrated the fibrosis formation and fiber orientation into the LA surface and enabled a virtual AF induction and AF wave-dynamic changes by using the dominant frequency (DF) and maximal slope of the action potential duration restitution curve (Smax; Lim et al., 2017). The fiber orientations were defined in the meshes of each patient’s geometry and adjusted based on the clinical local activation time map (Pashakhanloo et al., 2016). Bipolar voltage data obtained from catheter ablation mapping were matched onto the computational nodes of the LA 3D model, and the locations of the fibrotic areas were determined using the map (Figure 1B). The fibrosis status of each node was numerically defined (Hwang et al., 2019). We simulated the clinical local activation data by using the model, which reflected the cardiac structural orientation and fiber orientation (Figure 1C). The conductivity of the model was based on the status and shape of the fibrosis (Zahid et al., 2016). For the ion currents of fibrotic cells, the inward rectifier potassium current (I_K1_), L-type calcium current (I_CaL_), and sodium current (I_Na_) were decreased by 50, 50, and 40%, respectively, as compared to normal cells (Zahid et al., 2016). The reaction–diffusion equation for the cardiac wave propagation was solved numerically and adjusted based on the specific conduction velocity in each case to represent personalized AF simulations (Lee et al., 2016).

### AF Simulation

Figure 1F shows the process used in the study protocol. We induced AF in each case using AF pacing from 200 to 120 ms with eight beats per cycle lasting a total of 11,520 ms, based on appropriate ion current settings. Each virtual pacing location corresponded to the clinical activation time map for a realistic LA modeling, and the pacing sites were matched precisely to reflect the personalized LA models. AF maintenance was observed for 32 s. We defined a successful AF induction according to the electrograms in each LA model. AF defragmentation involved AF termination and AF conversion to atrial tachycardia.

### Virtual Interventions

We applied a virtual ablation and virtual anti-arrhythmia drug to our realistic AF model. For the virtual ablation, the membrane potential of the ablated regions was set as zero to produce a permanent conduction block interrupting the cardiac wave propagation. First, we performed a virtual PV isolation with four gaps located on the anterior and posterior sides of each pulmonary vein isolation line (Figure 1D). Then we ablated all those gaps to perform a circumferential pulmonary vein isolation. The size of each gap was 2 mm. We initiated AF induction after performing each ablation. We applied high dose virtual flecainide (15 μm) to the failed AF defragmentation cases. With the Courtemanche-Ramirez-Nattel model (Courtemanche et al., 1998; Sossalla et al., 2010; Grandi et al., 2011) as the baseline, the effects of flecainide were implemented by applying the percent changes for the specific ion channels. Compared with baseline AF, the I_to_, I_CaL_, I_Kr_, and I_Na_ were decreased by 5, 5, 30 and 45%, respectively (Wang et al., 1993; Hilliard et al., 2010; Kramer et al., 2013; Crumb et al., 2016; Geng et al., 2018; Sutanto et al., 2019).

### Analysis of the Spatial Changes in the AF Wave-Dynamics

We analyzed the wave-dynamics of the DF and Smax from 17 to 23 s. The DF was defined as the frequency with the highest power. It was analyzed using a Fourier transform for 6 s of action potentials at each node and the power spectra density function (Lim et al., 2017). We calculated the DF values for all nodes in the 3D LA model (Figure 1E). To examine the stability of the DF after each intervention, the coefficient of variation (COV) of the high-DF was calculated as the standard deviation divided by the mean. The Smax values were defined at every node in the LA regions of each patient.

### Statistical Analysis

The continuous variables are represented as the median and range. The proportion of categorical variables was compared among the groups using a Fisher’s exact test. Comparisons of the DF, Smax, and COV-DF were conducted using t-testing or Mann–Whitney testing, depending on the distribution. A value of *p* <0.05 was considered statistically significant. Statistical analyses were conducted using SPSS (Statistical Package for Social Sciences, Chicago, IL, United States) software for Windows (version 26.0).

## Results

### Wave-Dynamic Changes After the PVI

We induced virtual AF in realistic computational models obtained from 40 patients (Supplementary Table S1). PV interventions (40 CPVI and 40 PVI-gap) significantly increased the mean AF cycle-lengths (140.9 ms [129.9, 153.3] to 147.1 ms [131.6, 200.3], *p* = 0.045), but they did not change the mean DF (7.69 Hz [7.31, 8.47] to 7.65 Hz [6.27, 8.30], *p* = 0.150) or Smax (0.97 [0.81, 1.26] to 0.88 [0.66, 1.13], *p* = 0.144). The AF termination and defragmentation rates after the PV interventions were 20 and 46.3%, respectively. Any episode terminated before 17 s was excluded from the AF cycle-length, DF, and Smax analyses.

### Effects of the CPVI vs. PVI-Gaps on the Extra-PV Area

**Figure 2:**
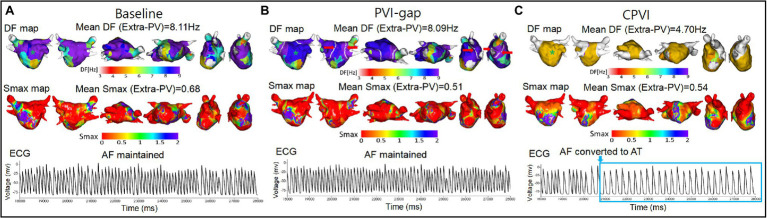
Wave-dynamic changes after the virtual PVI-gap and CPVI. **(A)** The ECGs were obtained at the blue ^*^ sites in the DF maps. **(B)** The red arrows indicate gaps in the PVI. **(C)** The 3D DF maps and ECGs indicate that the CPVI reduced the mean DF, increased the AF cycle lengths, and defragmented the AF, but the PVI-gap did not. However, neither the CPVI nor PVI-gap changed the Smax. DF indicates dominant frequency; Smax, the maximal slope of the restitution curves; PVI-gap, pulmonary vein isolation with gaps; CPVI, complete pulmonary vein isolation; AF, atrial fibrillation; AT, atrial tachycardia; ECG, electrocardiogram.

Table 1 compares the AF wave-dynamic changes between the CPVI and PVI-gap procedures. Compared with baseline AF, the CPVI significantly increased the mean AF cycle-lengths (*p* < 0.001), but the PVI-gap did not (*p* = 0.581). The CPVI also reduced the mean DF (*p* < 0.001), but the PVI-gap did not (*p* = 0.354, Figure 2). The amount of the DF reduction was significantly greater in the CPVI group than PVI-gap group (*p* < 0.001). The COV-DF, which reflected the spatial instability of the DF, was significantly greater in the CPVI group than PVI-gap group (*p* < 0.001). However, neither the CPVI (*p* = 0.445) nor PVI-gap (*p* = 0.078) changed the mean Smax (Table 1).

**Table 1:** Wave-dynamic changes and defragmentation after virtual PV interventions.

	Baseline	PVI-gap	CPVI	*p* value
	(*n* = 40)	(*n* = 40)	(*n* = 40)	
Mean AFCL	140.87 [129.87,153.26]	138.89 [128.21,149.25]	212.77^*^^,^^†^ [148.76,242.45]	<0.001
Mean DF (Hz)	7.690 [7.306,8.474]	8.061 [7.566,8.524]	5.760^*^^,^^†^ [4.984,7.847]	<0.001
ΔMean DF	NA	0.171 [−0.099,0.441]	−1.482^†^ [−3.184,−0.044]	NA
COV-DF (%)	NA	2.026 [1.190,4.555]	17.162^†^ [1.849,34.705]	NA
Mean Smax	0.974 [0.805,1.259]	0.847 [0.643,1.047]	0.943 [0.707,1.304]	0.208
ΔMean Smax	NA	−0.154 [−0.350,−0.034]	−0.102 [−0.350,0.181]	NA
Defragmentation, % (*n*)	0%(0/40)	12.5%(5/40)	80.0%(32/40)^*^^,^^†^	<0.001
Termination, % (*n*)	0%(0/40)	7.5%(3/40)	32.5%(13/40)^*^^,^^‡^	<0.001
Converted to AT, % (*n*)	0%(0/40)	5.0%(2/40)	47.5%(19/40)^*^^,^^†^	<0.001

*Smax, The maximal slope of the restitution curves; DF, Dominant frequency; COV-DF, Coefficient of variation-dominant frequency; AFCL, Atrial fibrillation cycle lengths*. *The median (IQ1, IQ3) is displayed in the table*.

* *p < 0.001* vs. *Baseline*.

† *p < 0.001* vs. *PVI-gap.*

‡ *p = 0.010* vs. *PVI-gap*.

### Defragmentation Rates After the CPVI vs. PVI-Gap and Flecainide

The AF defragmentation and termination rates after the PVI are listed in Table 1. There was no AF termination or defragmentation during the 32 s waiting period during the baseline AF. The AF termination rate after the CPVI was 32.5%, and it was 7.5% after the PVI-gap (*p* = 0.010). The AF defragmentation rates were also higher in the CPVI group (80.0%) than PVI-gap group (5%, *p* < 0.001). This finding was consistent after changing the location and number of PVI-gaps (Supplementary Table S2).

We added virtual flecainide to the failed defragmented episodes after the CPVI (*n* = 8) and PVI-gap (*n* = 35). The post-flecainide termination rate in the group after the CPVI (25.0%) tended to be higher than that in the PVI-gap group without statistical significance (2.9%, *p* = 0.084, Table 2).

**Table 2:** Flecainide-induced defragmentation rates of ongoing AF after the CPVI vs. PVI-gap.

	PVI-gap	CPVI	*p* value
	(*n* = 35)	(*n* = 8)	
Defragmentation, % (*n*)	25.7%(9/35)	62.5%(5/8)	0.089
Termination, % (*n*)	2.9%(1/35)	25.0%(2/8)	0.084
Converted to AT, % (*n*)	22.9%(8/35)	37.5%(3/8)	0.401

*PVI-gap, Pulmonary vein isolation with gap; CPVI, Complete pulmonary vein isolation; AT, Atrial tachycardia*.

### Mechanism of the AF Maintenance After the PVI-Gap

**Figure 3:**
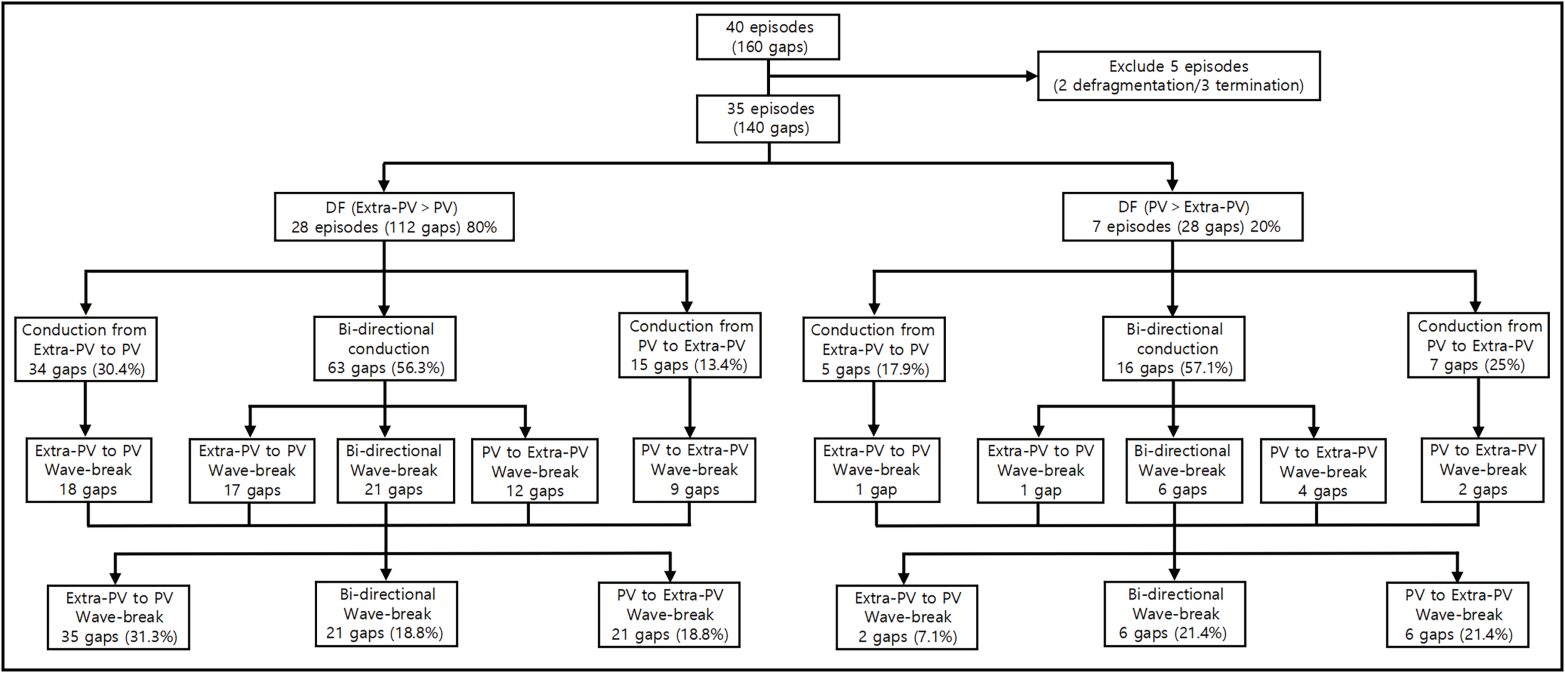
Wave-dynamic interactions between the PVs and extra-PVs in 35 ongoing AF episodes after the PVI-gap intervention. DF indicates dominant frequency; PV, pulmonary vein.

**Figure 4:**
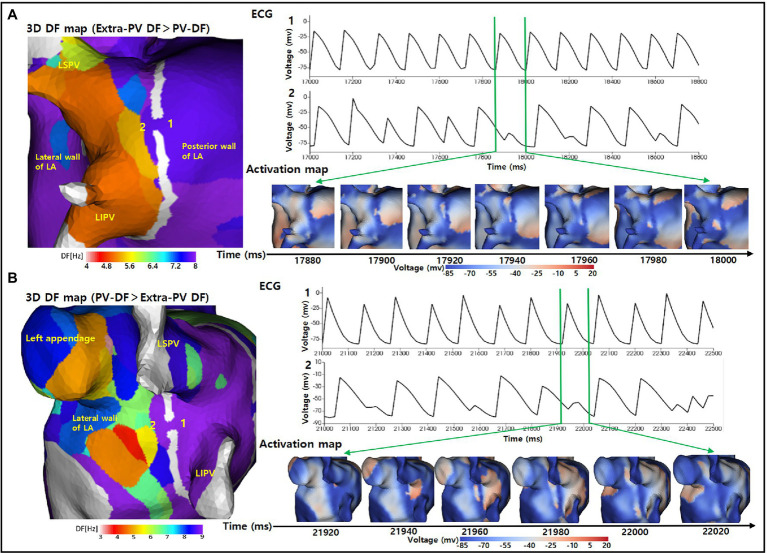
Wave-break at the wavelet exit of the gaps by a source-sink mismatch. **(A)** The 3D DF map indicates that the extra-PV DFs was greater than the PV-DFs. The activation map and ECG indicate that a regular wavelet turned into a wave-break when moving from the posterior wall of the LA to inside the PV through the gap. **(B)** The 3D DF map indicates that the inside PV-DF was greater than the extra-PV DF. The activation map and ECG indicate that a regular wavelet turned into a wave-break when moving from inside the PV to the lateral wall of the LA through the gap. DF indicates dominant frequency; PV, pulmonary vein; LA, left atrium; LSPV, left superior pulmonary vein; LIPV, left inferior pulmonary vein; ECG, Electrocardiogram.

We evaluated the PV and extra-PV wave-dynamic interactions in 35 ongoing AF episodes after the PVI-gap intervention (Figure 3). The extra-PV DF was greater than the PV-DF in 28 of those episodes (80%), and the inside PV-DF was greater than the extra-PV DF in 7 episodes (20%). The wavelet interactions were maintained through the PVI-gaps for as long as the AF was maintained. The wave-breaks generally appeared at the wavelet exit of the gaps by a source-sink mismatch (Figure 4). The rate of a wave-break did not differ between the episodes in which the wavelet moved from the higher DF site to the lower DF site and that in which it moved from the lower DF site to the higher DF site (48.8% vs. 50%, *p* = 1.000).

**Figure 5:**
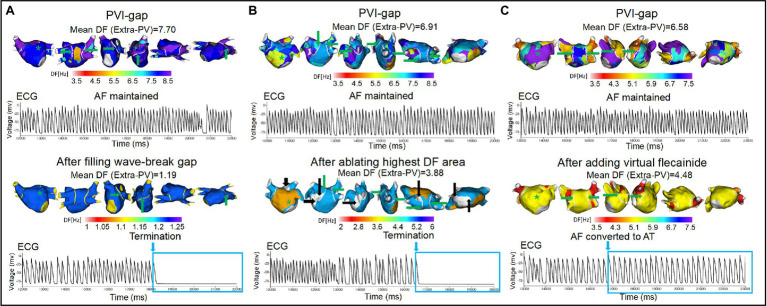
Wave-dynamic changes after filling the wave-breaking PVI-gaps, ablating the highest DF sites, and adding virtual flecainide. ECGs were obtained at the green ^*^ sites in the DF map, and the green arrows indicate the gaps in the PVI. **(A)** AF terminated after ablating the two gaps on the posterior wall of the LA. **(B)** AF terminated after ablating the high DF site (white areas indicated by black arrows). **(C)** AF converted to AT after adding virtual flecainide without additional ablation. PVI-gap indicates pulmonary vein isolation with gaps; PV, pulmonary vein; DF, dominant frequency; AF, atrial fibrillation; AT, atrial tachycardia; ECG, Electrocardiogram.

Among the 35 episodes of ongoing AF after the PVI-gap, filling the wave-breaking PVI-gaps (total 91 gaps), ablation of the highest DF site without touching the PVI-gaps (112 extra-PV and 51 intra-PV sites), and additional virtual flecainide defragmented the AF in 60.0, 34.3, and 25.7% of cases, respectively (*p* = 0.010, Figure 5). The AF termination rate was significantly greater after filling the PVI-gaps than after ablating the highest DF or administering flecainide (*p* = 0.010, Table 3).

**Table 3:** Defragmentation rates after interventions for 35 AF PVI-gap episodes.

	Wave-break gap ablation	Highest DF sites ablation	Add virtual flecainide	*P* value
	(*n* = 35)	(*n* = 35)	(*n* = 35)	
AF Defragmentation, % (*n*)	60.0%(21/35)^*^	34.3%(12/35)	25.7%(9/35)	0.010
AF termination, % (*n*)	25.7%(9/35)^†^	8.6%(3/35)	2.9%(1/35)	0.010
AF converted to AT, % (*n*)	34.3%(12/35)	25.7%(9/35)	22.9%(8/35)	0.538

*AF, Atrial fibrillation; DF, Dominant frequency; AT, Atrial tachycardia*.

* *p = 0.007* vs. *Add virtual flecainide*.

† *p = 0.013* vs. *Add virtual flecainide*.

## Discussion

### Main Findings

In this study, we used realistic human AF computational modeling to explore how a CPVI affected the extra-PV wave-dynamics and PVI-gaps as a mechanism for AF recurrence. The CPVI significantly reduced the mean extra-PV DF and its spatial stability (increased COV-DF), but it had no effect on the Smax, an index of wave-breakability. In the episodes of ongoing AF after the PVI-gap, wave-breaks commonly occurred at the wavelet exit of the gaps. Additional ablation of wave-breaking PVI-gaps had a greater defragmentation effect than extra-PV DF ablation or virtual flecainide. Therefore, the CPVI effectively reduced the mean DF in the extra-PV area, and under the PVI-gap condition, filling the PVI-gaps had anti-AF effects superior to those of an extra-PV DF ablation or additional flecainide.

### Extra-PV Effects of the CPVI

The CPVI has traditionally been the cornerstone of AFCA (Oral et al., 2006), and it has several potential anti-AF mechanisms. First, the CPVI blocks the PV triggers (Oral et al., 2006). Second, the CPVI partially denervates cardiac autonomic nerves, including the ganglionated plexi around the PV antrum (Po et al., 2009). Third, a wide CPVI significantly reduces the atrial critical mass (Hwang et al., 2012). We have here identified another novel mechanism by which the CPVI significantly reduces the mean DF and spatial heterogeneity of the DF at extra-PV sites. Among the 40 patients who were included in this study, 93% had persistent AF, and we integrated their voltage-activation maps into our realistic computational models. We found that the CPVI still plays an important role in the extra-PV AF maintenance mechanism, even in AF patients with atrial substrate remodeling. To date, no empirical extra-PV LA ablation, including linear, electrogram-guided, or rotor ablation, has shown equivalent rhythm outcomes in patients with a CPVI, despite multiple randomized clinical trials (Verma et al., 2015). Our results reconfirm the importance of a CPVI in the LA, even though extra-PV areas, including the right atrium, contribute to the maintenance mechanisms of AF (Lim et al., 2020b).

### Role of PV-Gaps as a Mechanism of Recurrence After a PVI

Irrespective of the uncertain unifying mechanisms of AF, a PV electrical isolation is an objective, standard, and widely accepted minimal requirement for AFCA (Calkins et al., 2012). However, despite advances in the catheter efficiency, a long-term durable PVI still has technical limitations. The rate of PV reconnections during a repeat ablation has been reported to range from 36% to more than 95% in repeat procedures (Lin et al., 2015). Many studies have reported that PV reconnections are the leading cause of arrhythmia recurrences. However, PV reconnections have also been observed in patients without AF recurrence (Nilsson et al., 2006). In this study, we evaluated how PV gaps affected the AF maintenance mechanisms. Of the four 2-mm PV gaps that remained in our model, 65% (91/140) were accompanied by wave-breaks and 19.3% (27/140) contributed to wave-breaks in the PV to LA direction. The PV gap size also had an essential influence on the AF maintenance. Following Herweg’s report, we applied 2-mm gaps, which were suitable for generating wave-breaks by a source-sink mismatch (Herweg et al., 2021).

### Appropriate Management of Recurrent AF After AFCA

The most effective repeat procedures for patients with recurrence after AFCA is not yet known. Depending on the recurrent AF burden or the operators’ discretion, gap fillings for the reconnected PVs or an empirical extra-PV ablation, such as a posterior wall isolation, have been performed (Lee et al., 2019). In patients with a well-maintained PVI during redo-mapping, extra-PV triggers play a significant role in AF recurrence, and the outcomes of a repeat ablation are worse than in patients with PV reconnections (Kim et al., 2021). In this study, we examined the AF maintenance mechanism during the PVI-gap state by using computational models made with the electroanatomical maps of 40 patients, most of whom had persistent AF. Under those controlled conditions, filling the wave-breaking gap produced a more effective AF defragmentation than ablation of the extra-PV DF sites or using antiarrhythmic drugs. That was consistent with the results of a recent randomized clinical trial, suggesting that a durable PVI is more effective than an empirical extra-PV ablation as a repeat AF ablation procedure (Kim et al., 2022).

### Limitations

This study had some limitations in its computational simulations. First, bi-atrial modeling manifesting interatrial conduction was not applied in this study. Second, we did not consider the LA wall thickness in our LA model. Third, because we applied fibrosis based on voltage-map, not a magnetic resonance image (Boyle et al., 2019; Baek et al., 2021), it is not clear whether the detailed structure of cardiac fibrosis with microchannels is adequately reflected in our morel. Fourth, our personalized LA model consisted of a monolayer, not bilayers representing the endocardial and epicardial layers. Fifth, the rate-dependent effect of flecainide on I_Na_ may not be reflected sufficiently in this study (Moreno et al., 2011).

### Conclusion

The CPVI effectively reduced the mean DF and increased its spatial heterogeneity in the extra-PV areas. Filling the PVI-gaps had anti-AF effects superior to those of an extra-PV DF ablation or additional flecainide under the PVI-gap condition.

## Data Availability Statement

The original contributions presented in the study are included in the article/Supplementary Material, further inquiries can be directed to the corresponding author.

## Ethics Statement

The studies involving human participants were reviewed and approved by Institutional Review Board of Severance Cardiovascular Hospital, Yonsei University Health System. The patients/participants provided their written informed consent to participate in this study.

## Author Contributions

ZJ contributed to the data, statistical analyses, and writing of the manuscript. IH contributed to the statistical analyses and data acquisition. O-SK contributed to the software programming and data acquisition. BL confirmed the data acquisition and references. J-WP contributed the clinical data acquisition. H-TY, T-HK, BJ, and M-HL contributed to the clinical data acquisition and interpretation of clinical data. H-NP contributed to the study design, clinical data acquisition, data interpretation, and revision of manuscript. All authors contributed to the article and approved the submitted version.

## Funding

This work was supported by grants (HI19C0114) and (H21C0011) from the Ministry of Health and Welfare and grants (NRF-2020R1A2B01001695) and (NRF-2019R1C1C1009075) from the Basic Science Research Program run by the National Research Foundation of Korea (NRF), which is funded by the Ministry of Science, ICT, and Future Planning (MSIP).

## Conflict of Interest

The authors declare that the research was conducted in the absence of any commercial or financial relationships that could be construed as a potential conflict of interest.

## Publisher’s Note

All claims expressed in this article are solely those of the authors and do not necessarily represent those of their affiliated organizations, or those of the publisher, the editors and the reviewers. Any product that may be evaluated in this article, or claim that may be made by its manufacturer, is not guaranteed or endorsed by the publisher.
