# Supplementary material for: Anti-atrial Fibrillation Effects of Pulmonary Vein Isolation With or Without Ablation Gaps: A Computational Modeling Study
Source: Front Physiol. 2022 Mar 17;13:846620. doi: 10.3389/fphys.2022.846620 (PMC8968313; doi:10.3389/fphys.2022.846620)
Supplement: Supplementary file 1 [file Table_1.DOCX]

**Supplemental Table S1.** Patient characteristics

| **Characteristics** | **N=40** |
| --- | --- |
| Age, years | 61.0±9.8 |
| >75 years old | 2 (5.0%) |
| 65-75 years old | 23 (57.5%) |
| <65 years old | 15 (37.5%) |
| Gender |  |
| Male | 32 (80.0%) |
| Female | 8 (20.0%) |
| Persistent AF | 37 (92.5%) |
| CHA_2_DS_2_-VASc score | 2.20±1.62 |
| Heart failure | 12 (30.0%) |
| Hypertension | 21 (52.5%) |
| Diabetes | 7 (17.5%) |
| Previous stroke | 9 (22.5%) |
| Previous TIA* | 0 (0%) |
| Vascular disease | 2 (5%) |
| Echocardiographic findings |  |
| Left atrium dimension | 44.6±5.0mm |
| Ejection | 60.5±7.8% |
| E/Em | 10.4±3.8 |

AF, atrial fibrillation; TIA, transient ischemic attack; LA, left atrium; EF, ejection fraction; E/Em, the ratio of the early diastolic mitral inflow velocity (E) to the early diastolic mitral annular velocity (Em).

**Supplemental Table S2.** Defragmentation rates after different location and number of PVI-gaps.

|  | Baseline | PVI-4 gap  (2 anterior + 2 posterior) | PVI-4 gap  (2 inferior + 2 superior) | PVI-8 gap  (2 anterior + 2 posterior + 2 superior + 2 inferior) | CPVI | P value |
| --- | --- | --- | --- | --- | --- | --- |
| Defragmentation, % (n) | 0% (0/40) | 12.5% (5/40) | 15.0% (6/40) | 10.0% (4/40) | 80.0% (32/40)*† | <0.001 |
| Termination, % (n) | 0% (0/40) | 7.5% (3/40) | 2.5% (1/40) | 7.5% (3/40) | 32.5% (13/40)‡§ | <0.001 |
| Converted to AT, % (n) | 0% (0/40) | 5.0% (2/40) | 12.5% (5/40) | 2.5% (1/40) | 47.5% (19/40)†‡ | <0.001 |

PVI, Pulmonary vein isolation; CPVI, Complete pulmonary vein isolation.

*, p<0.001 vs. PVI-4 gap (2 inferior + 2 superior);

†, p<0.001 vs. PVI-8 gap (2 anterior + 2 posterior + 2 superior + 2 inferior);

‡, p=0.001 vs. PVI-4 gap (2 inferior + 2 superior);

§, p=0.010 vs. PVI-8 gap (2 anterior + 2 posterior + 2 superior + 2 inferior).
